# Common herbicide impairs fertility but not survival in bumblebees, *Bombus impatiens*

**DOI:** 10.1038/s41598-025-22720-w

**Published:** 2025-11-26

**Authors:** Andrew F. Brown, Pierre Giovenazzo, Marilène Paillard, Andrée Rousseau, Verena Strobl, Annette Van Oysteayen, Peter Neumann, Lars Straub

**Affiliations:** 1 Institute of Bee Health, Vetsuisse Faculty, University of Bern, Bern, Switzerland; 2 Department of Biology, Vachon Pavillon, Université Laval, Québec, QC G1V 0A6 Canada; 3 Centre de Recherche en Sciences Animales de Deschambault (CRSAD), Deschambault, QC Canada; 4 Swiss Bee Research Center, Agroscope, Bern, Switzerland; 5 Biobest Group NV, Research and Development, Westerlo, Belgium; 6 Department of Biological Sciences, Centre for Ecology, Evolution, and Behaviour, Royal Holloway University of London, Egham, UK

**Keywords:** Agrochemicals, Biodiversity, Fitness, Risk assessments, Reproductive toxicology, Sustainability, Developmental biology, Ecology, Ecology, Physiology, Zoology

## Abstract

Insect declines have raised concerns over widespread agrochemical usage. Current environmental risk assessments (ERAs) focus on lethal effects, often neglecting fitness-relevant parameters. Here, we experimentally exposed male bumblebees (*Bombus impatiens*) to a field-realistic concentration of a glyphosate-based herbicide (GBH) under controlled laboratory conditions to assess potential effects on survival, consumption behavior, and reproductive physiology (i.e., spermatozoa traits). Our results demonstrate that chronic exposure of male bumblebees to GBH significantly enhanced their survival yet reduced living spermatozoa (~ 34%). This highlights a physiological shift, where resources appear prioritized toward survival mechanisms over reproductive investment. Such findings underscore critical false negative results in current ERAs and advocate for incorporating fitness endpoints to better understand and mitigate ongoing insect declines.

**Supplementary Information:**

The online version contains supplementary material available at 10.1038/s41598-025-22720-w.

Agrochemicals have increasingly come under scrutiny for their inadvertent effects on non-targeted organisms^1^, prompting the development of regulatory frameworks designed to mitigate environmental hazards prior to market approval^2^. Emerging data highlight that sublethal impacts on fitness^3^—largely overlooked in pollinator risk assessments focused on mortality^2^—pose serious threats to wild insect populations. Toxicological studies reporting no significant impact or even increased survival may obscure underlying fitness costs due to a potential trade-off between survival and reproduction^3^. Such findings may lead to false-negatives and underestimating agrochemical risks in regulatory assessments. Therefore, it is critical that ecotoxicological risk assessments (ERAs) incorporate fitness-related endpoints to more accurately evaluate the risks posed by chemical exposures^4^.

In eusocial bumblebees (*Bombus* spp.), which typically exhibit monandrous mating systems^5^ (i.e., females mate with a single male), male reproductive capacities are essential for both individual as well as colony fitness. Males are critical for sustaining wild populations and their contribution depends on successfully reaching sexual maturity, locating a mate, and producing high quantities of viable spermatozoa^5^. Male bumblebees (i.e. drones) generally attain sexual maturity five days post-eclosion, after which a single copulation with a virgin female will determine her lifetime storage of spermatozoa and fecundity^5^. Studies revealing that insecticides, especially neonicotinoids, can impair essential male fitness components like spermatozoa traits^6^ are alarming, given the foundational role of male fertility in colony formation and broader population dynamics. Thus, xenobiotic-induced impairments of male fertility—reported across multiple taxa, including humans^7^—may represent a critical yet underrecognized key factor contributing to the persistent decline of insect populations^1^.

Glyphosate-based herbicides (GBHs) make up > 50% of the global pesticide market and are amongst the most widely used agrochemicals^8^. Although the metabolic-inhibitory effect of glyphosate is specific to plants and micro-organisms through inhibition of the shikimate pathway^9^, its chelating properties allow it to bind essential immune-related minerals (e.g. manganese), and non-target side effects have been reported in both invertebrates and vertebtrates^10^, including sensory (i.e. vision) impairment in bumblebees^11^. The presumed safety of glyphosate for bees and other insects has been largely inferred from toxicity tests that focus primarily on survival^12^. Field-relevant concentrations of both the active ingredient (glyphosate) and commercial formulations containing additives (i.e. adjuvants^13,14^) are often assumed to be non-lethal, and remain largely exempt from regulatory toxicity testing^15^. However, accumulating evidence highlights the critical role of adjuvants in driving toxicity, including for instance direct associations with reduced bumblebee survival^16^. This is further supported by a meta-analysis showing two-thirds of the evaluated studies reported greater harm to non-target organisms from formulated products than the active ingredient alone^17^. Nevertheless, the potential effects of GBH on male insect fertility, such as spermatozoa traits (e.g., quantity and viability), remain largely unexplored. Using a modified toxicity protocol from the Organization for Economic Co-operation and Development (OECD), we assessed the effects of a common field-realistic exposure scenario of a commercially available glyphosate-based product on male bumblebees, *Bombus* *impatiens*. Our data reveal a striking disconnect between survival metrics and male fertility, highlighting that current ERA frameworks substantially underestimate the ecological risk associated with GBH products to insect populations.

## Results

### Daily consumption and exposure

For males caged individually, sucrose consumption ranged from 0.0300–0.1633 g (*N* = 50, *μ* = 0.1081 g, *σ* = 0.0344 g, control) and 0.0367–0.1958 g (*N* = 50, *μ* = 0.1162 g, *σ* = 0.0378 g, herbicide). For males in hoarding cages, sucrose consumption ranged from 0.0537–0.1483 g (*N* = 54, *μ* = 0.0934 g, *σ* = 0.0199 g, control) and 0.0522–0.1110 g (*N* = 54, *μ* = 0.0843 g, *σ* = 0.0148 g, herbicide). No statistical difference between treatments of males held in individual cages was found (Extended Data Fig. S2A, *Ps* > 0.05). However, GBH-exposed males held in hoarding cages consumed ~ 9.6%. less than their respective control counterparts (Extended Data Table S1 & Fig. S2B, *P* < 0.05). Cumulative exposure of GBH for individual hoarding cages after three days was *μ* = 2,547 ng (*σ* = 870.68 ng) and *μ* = 9,840 ng (*σ* = 2,357.165 ng) after 10 days.

### Survival

**Fig. 1:**
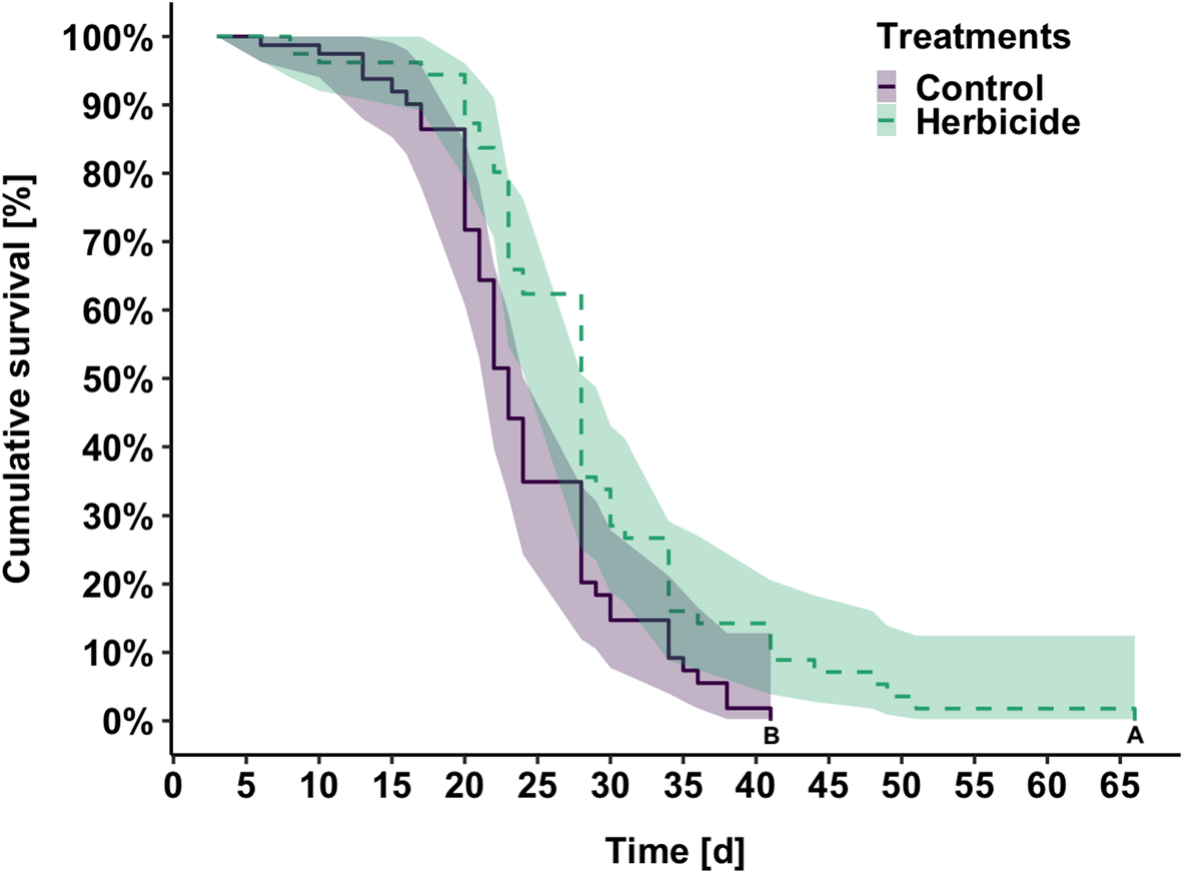
Kaplan Meier survival curves of male bumblebees, *Bombus impatiens*, from control (*N* = 104, violet) and herbicide treatments (*N* = 104, green). Shaded 95% Cis are shown, and letters denote statistical significance (*P* < 0.05).

Longevity ranged from 3–41 days (*N*_control_ = 104,), with a median age of 23 days (95% CI 22–28), and 3–66 days (*N*_herbicide_ = 104,) with a median age of 28 days (95%CI 28–29). Cage type did not influence survival (cox proportional hazards regression model, *P* > 0.05). As such, data was pool for Kaplan Meier analysis, and males exposed to GBH lived significantly longer than the controls (Fig. 1, χ^2^ = 9.3, *P* = 0.002).

### Total living spermatozoa

**Fig. 2:**
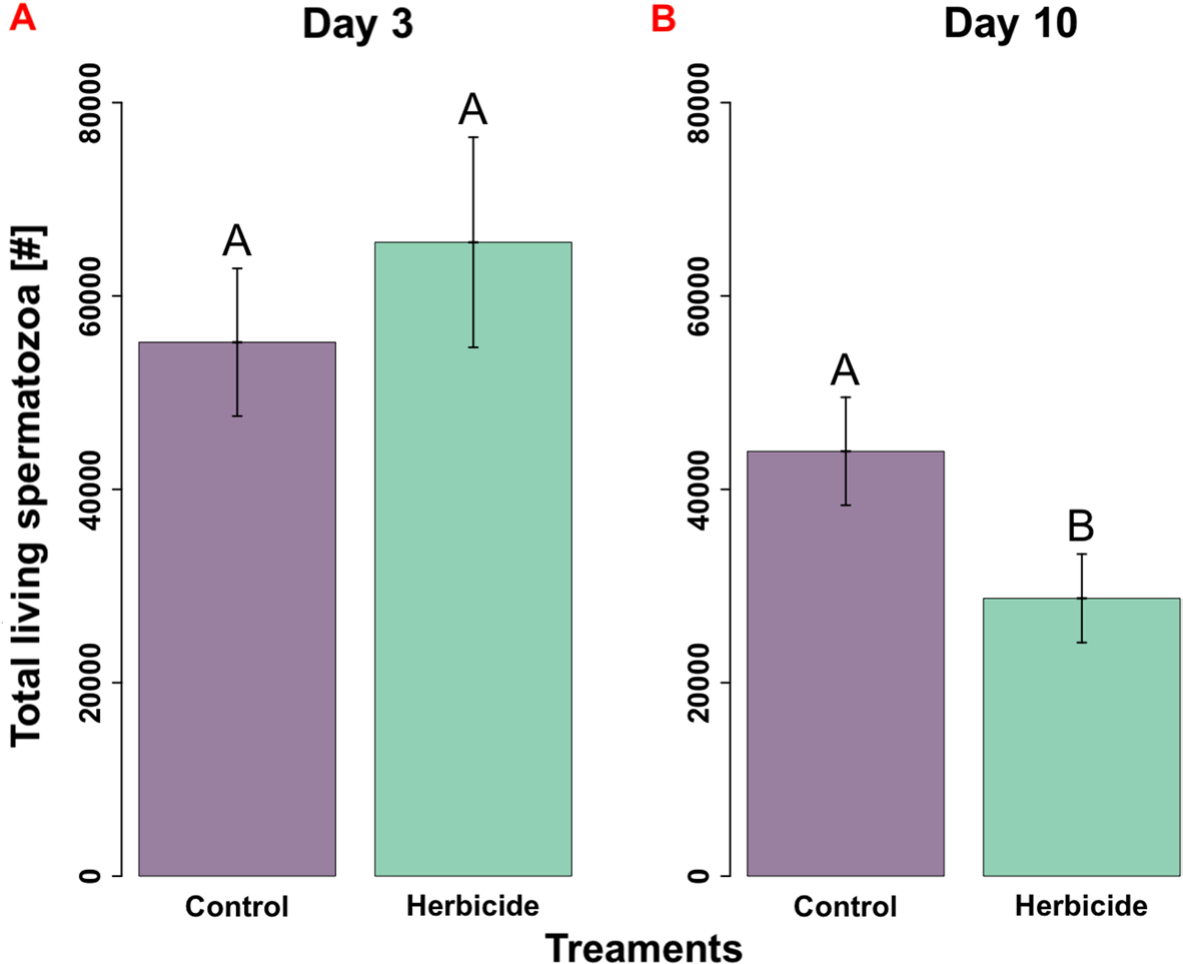
(**A, B**) : Bar charts of total living spermatozoa from *Bombus impatiens* males subject to control and herbicide treatments, split into 3 (*N* = *22* control, *N* = 24 herbicide, (**A**)) and 10 days (*N* = 19 control,* N* = 22 herbicide, (**B**)) of herbicide exposure. Means, standard error, and letters denoting statistical significance within an exposure time (*P* < 0.05) are shown.

Total number of living spermatozoa (TLS) did not significantly differ between the treatment groups on day three (*P* > 0.05; Fig. 2A), with a pooled mean of 60,606 (95% CI $\pm$ 13,525) TLS across both treatment groups (*N*_control_ = 22, *N*_herbicide_ = 24, *N*_total_ = 46). In contrast, the 10-day-exposure to GBH significantly reduced the TLS ($\mu \hspace{0.17em}$= 28,730 TLS, 95%CI ± 9512) compared to the control ($\mu$ = 43,938 TLS, 95% CI $\pm$ 11,732) (*P* = 0.04213; Fig. 2B), resulting in exposed males having ~ 34% fewer living spermatozoa (*N*_control_ = 19, *N*_herbicide_ = 22, *N*_total_ = 41). Further, a significant negative correlation (*R* = − 0.44, *P* = 0.003) was observed between TLS and GBH exposure (Extended Data Fig. S3), where increasing exposure led to fewer TLS.

## Discussion

Our data clearly show for the first time that exposure to a GBH can significantly impair male insect fertility. Despite exhibiting increased longevity, potentially indicative of pesticide-induced hormesis^18^, chronically exposed bumblebee males showed a ~ 34% reduction in living spermatozoa compared to controls. These findings suggest a potential trade-off between survival and reproductive capacities^18^, offering a plausible mechanistic explanation for the ongoing decline in entomofauna and highlight a critical limitation of current ERAs. Namely that the prevailing reliance on lethal endpoints in ecotoxicological testing^2^ underestimates the true ecological risks and increases the likelihood of false-negative outcomes if fitness relevant traits are systematically overlooked^4^. A fundamental paradigm shift in conventional ERAs is urgently needed that integrates fitness as a key endpoint and explicitly includes male insect reproductive health to more accurately assess the impact of agrochemical exposure.

Although 10-day oral chronic toxicity assays are standard practice in ecotoxicology^2^, such short-term designs would have overlooked the findings of altered consumption behaviour and extended survival observed over the full life cycle. Indeed, the lack of feeding impairment during exposure aligns with prior studies^19^, however, the emergence of behavioural changes post-exposure suggests underlying impairments in cognitive functions, such as learning and memory, and/or disruptions to neurotransmitter signalling^19^—essential for effective foraging and nutrient uptake. Albeit the risk of malnutrition in exposed bees cannot be formally ruled out, all treatments were given uniform access to pollen, and the survival data do not support negative nutrition-related effects. Moreover, inadequate nutrient intake is unlikely to have influenced spermatozoa traits, which were assessed during the exposure period where consumption was not affected. Despite previous studies having reported increased longevity in honeybees following GBH exposure^20^, the majority have documented neutral or adverse effects on survival^19^. The primary mechanistic explanation links glyphosate to disruption of the gut microbiota and impaired immune function^21^. Glyphosate inhibits microbial enzymes, causing dysbiosis—particularly a reduction of beneficial bacteria such as *Snodgrassella alvi*—which increases mortality risk and susceptibility to pathogens and other stressors^12,20^. Furthermore, glyphosate has been shown to dysregulate immune responses by downregulating antimicrobial peptide expression (e.g., defensin, apidaecin, hymenoptaecin) as well as melanization pathway inhibition, thereby potentially compromising infection resistance^22^. On the other hand, the observed increased in survival may reflect a biphasic (hormetic) response^23^, including the upregulation of detoxification enzymes (e.g., cytochrome P450s^24^) and enhanced antioxidant expression^25^, which can modulate detoxification and metabolic pathways. Nonetheless, laboratory findings may not necessarily reflect real-world scenarios, as controlled laboratory conditions cannot capture the complexity of natural environments^13^. Therefore, it is essential to validate these results under full field or a least seme-field conditions^26,27^, where bumblebees perform energetically demanding tasks, such as foraging. Such activities may interact with GBH exposure antagonistically, additively, and/or synergistically, thereby altering the observed laboratory outcomes^11,28,29^. Regardless of the underlying mechanisms, increased survival likely entails physiological cost, potentially manifesting with fitness-related proxies^30^. Indeed, numerous studies report altered fitness reproductive traits following pesticide exposure, despite no direct effect on survival^20,31,32^.

Prolonged exposure to GBH led to a ~ 34% reduction in total living spermatozoa in male bumblebees. To the best of our knowledge, this is the first report of such an effect induced by a GBH in insects. While our study does not disentangle the effects of the active ingredient glyphosate and those of co-formulants (e.g., adjuvents^13^), similar impairments have been documented in vertebrates, including mammals, following exposure to both isolated glyphosate and GBH formulations^33,34^. Potential mechanisms include endocrine disruption (e.g. hormone deregulation^35^), oxidative stress (e.g. cytotoxicity increase^36^), and interference with energy metabolism (e.g. depressed ATP synthase activity^37^), processes widely implicated in reproductive dysfunction^38^. Additionally, pesticide-induced dysbiosis, particularly following cumulative GBH exposure (i.e., > 5 days^20^), can disrupt gut microbiota critical to host health, and notably, similar symbionts have recently been identified in bee seminal vesicles^39^. These gradual microbial shifts may impair reproductive function and spermatozoa quality, highlighting a potential novel pathway by which GBH could compromise male fertility. Irrespective of the mechanism, impaired male fertility will inevitably reduce fitness in both male and female bumblebees. Given that bumblebee males are polygynous (i.e., can mate with multiple females^5^), decreased spermatozoa quantity will limit their mating potential. Further reduced spermatozoa viability may result in more unintentional males emerging from unfertilized eggs due to haploid-induced sex determination in hymenoptera^40^. As bumblebee queens are typically single-mated, coupled with males that do not help with colony duties^41^, such unintentional sex imbalances (i.e. male-biased ratios) could lead to reduced colony functionality, with downstream effects on future population dynamics and production of new queens.

In line with increasing evidence that agrochemicals impair male fertility across taxa^3,42^, and considering past neonicotinoid data^6^ as well as widespread environmental pesticide contaminations^1^, our results strongly suggest that male insect infertility may indeed represent a widespread yet vastly overlooked consequence of anthropogenic chemical exposure. Further, the data underscore the risk of drawing false negative conclusions when relying primarily on lethality, reinforcing the need to incorporate fitness-related measurements into pollinator risk assessments^4^. Spermatozoa traits in male insects are readily quantifiable and, in bumblebees, can be assessed using modified acute toxicity protocols^2^, offering a practical and ecologically relevant framework for evaluating potential agrochemical hazards. In conclusion, a paradigm shift in ERAs is imperative, one that includes reproductive fitness parameters, to more accurately evaluate the ecological impact of agrochemicals and address the ongoing decline of the global entomofauna^29^.

## Methods

The study was performed at the Centre de recherche en sciences animales de Deschambault, Québec, Canada. To establish known age-cohorts, 220 newly emerged *Bombus impatiens* males were randomly collected from over 30 colonies by Biobest Canada Ltd., Ontario, Canada. Age was determined based on their physical appearance (i.e. *Bombus* spp. aged 0–24 h have silvery grey coloration phenotype indicating recent eclosion^43^). Visual inspections revealed that all individuals were free of ectoparasite infestations, clinical symptoms of disease, and other abnormalities^41^. Nevertheless, twelve randomly selected individuals were dissected following standard protocols^44^ and gut tissues were examined under light microscopy to confirm the absence of gut parasites (i.e., *Crithidia bombi, Nosema bombi, and Apicystis bombi*).

### Set up

A total of 208 male bumblebees were individually weighed (± 0.01 mg) using a precision balance (Kilotech KHA 203, Montreal, Canada), and subsequently randomly allocated to either individual cages^4^ (volume: 80 cm^3^; Extended Data Fig. 1A) or group hoarding cages (volume: 100 cm^3^; six individuals per cage; Extended Data Fig. 1B). Each cage received a 5 mL syringe (Codan Medical AG, Switzerland) of sucrose solution (1:1 [w/w]) which was weighed (± 0.01 mg) to later account for consumption behavior. Following OECD guidelines, evaporation was adjusted for by placing syringes in five empty cages, revealing negligible evaporation (< 1%). In addition, each cage was provided with *ad libitum* access to an Eppendorf® tube containing pollen paste (60% fresh honeybee-collected corbicular pollen provided by a local organic beekeeper, and 40% sugar powder) to enable tissue and organ development^45^. Cages were then assigned to one of the two treatment groups: Controls (sucrose solution; *N* = 104) and herbicide (herbicide spiked sucrose solution; *N* = 104 drones). Within each treatment group, 50 bees were caged individually (i.e., *N*_individual cages_ = 50) and 54 were caged in hoardings cages consisting of six individuals (*N*_hoarding cages_ = 9). All cages were placed into an incubator at 25 °C ± 1 °C and 50% RH ± 10% in complete darkness^46^.

### Chronic exposure and herbicide solution preparation

A commercially available glyphosate-based herbicide (GBH) product, Roundup Weather Max®, was used (Glyphosate = 540 g acid equivalent per Liter, present as potassium salt; Monsanto Canada Inc., Winnipeg, Canada). The GHB was dissolved in sugar water (1:1 [w/w]) to receive a field realistic concentration of 7.6 mg a.i.L^−1^^47,48^. All males in the GBH treatment group were chronically exposed to the herbicide for ten days, unless they died during the exposure period or were sampled for spermatozoa trait assessments at day 3 (*N*_control_ = 22, *N*_herbicide_ = 24) in which case exposure lasted three days. Thereafter, the individuals from the GBH-treatment received control sucrose solution for the remaining duration of the experiment (i.e., until all specimens died).

### Consumption, exposure, and survival

Pollen consumption was not measured due to males moving pollen throughout the cages (as observed in Brandt et al.^49^), thus all consumption data refers to sucrose consumption. Consumption was measured by recording the mass of the syringe on day 0, day 3, day 10, and/or at the point of death of a specimen. For the hoarding cages, consumption was additionally divided by the number of bees alive at any given timepoint to obtain consumption per individual bee. Total consumption [g] was divided by the number of days that the bees were alive to obtain an average daily consumption [g day^−1^]. Daily and total consumption were then multiplied by the exposure concentration to calculate daily [ng day^−1^] and total [ng] herbicide exposure for each specimen, respectively. Survival was recorded every 24 h until the last bee had died.

### Spermatozoa quantity and viability

To confirm that males were not sexually mature at the onset of the experiment, a subsample of individuals (*N* = 12) was dissected upon arrival and examined for the presence of spermatozoa. No spermatozoa were detected in the seminal vesicles, where they are typically found following the onset of sexual maturity, confirming the immaturity of all assessed specimens^5^. Only surviving bees from the individual cages were assessed for spermatozoa quantity and viability on days three and 10 following the initiation of the cage assay (day 3: *N*_controls_ = 22, *N*_herbicide_ = 24 and day 10: *N*_controls_ = 19, *N*_herbicide_ = 22). These individuals were selected because their exposure scenarios were precisely known due to the individual consumption value. Subsequently, males were between five and six days of age and considered sexually mature upon the first measurement (emergence time + 48-h shipment + 72-h exposure). The second measurement was performed to assess spermatozoa quantity and viability after 10 days of chronic GBH exposure^50,51^.

Individuals were briefly anaesthetized using CO_2,_ pinned to a wax plate, and dissected. Spermatozoa samples were collected from live bees following a modified protocol from Baer and Schmid-Hempel^50^. Here, the entire male genitalia, including the granular gland, accessory gland, vesical seminalis and testis were removed from each male, placed in a 1.5 mL Eppendorf® tube containing 250 μL Kiev + buffer, and gently crushed to form a diluted spermatozoa stock solution. Immediately following homogenization, a 50 μL aliquot of the spermatozoa stock solution was set aside in a separate 1.5 mL Eppendorf® tube for analyses of spermatozoa viability (proportion of living spermatozoa). Spermatozoa viability and quantification was done according to established protocols^42,51^. Lastly, the total living spermatozoa was calculated as the product of spermatozoa quantity and viability, as described in Straub et al.^52^. Full details can be found in Extended Data: Spermatozoa sample preparation.

### Data analyses

All data were analysed with R^53^, and when appropriate, data distributions were verified visually^54^ combined with Lilliefors^55^ (Kolmogorov–Smirnov) normality tests (*P*s > 0.05). All statistical code is accessible through the Dryad Repository (see “Data Availability”). Descriptive statistics, including means (μ), standard deviations (σ), medians, and 95% confidence intervals (CI), are reported where applicable. Briefly, daily consumption data were normally distributed (*P* > 0.05) and statistical differences between cage types and treatment groups were determined using Welch t-tests (non-paired, $\propto =0.05)$. Survival was assessed using Cox Proportional Hazard Regression models and Kaplan–Meier survival estimates were performed with the survival^56,57^ and survminer^58^ packages, and χ^2^ tests of equality were calculated with the Surfdiff() function (rho = 0). Total living spermatozoa was assessed in two separate increments: (1) three-days of exposure (five-day-old males) and (2) and ten-days of exposure (12-day-old males). Subsequently, two least-squares regression models (*lm*) for each timepoint were done, each with a Gaussian distribution due to normality assessments (*P* > 0.05). Total living spermatozoa (response variable) was modelled as a function of treatment.

To test for significant differences, Welch t-tests were performed (non-paired, $\propto =0.05)$.

A complementary least-squared model and correlation analysis (Pearson) was run in parallel replacing the treatment factor with a continuous exposure variable to assess dose-dependent effects (Extended Data Fig. S2):

## Supplementary Information

Below is the link to the electronic supplementary material.

Supplementary Material 1

## Data Availability

The raw data and statistical code are available on the Dryad repository: 10.5061/dryad.n02v6wx8s
