# Supplementary material for: Common herbicide impairs fertility but not survival in bumblebees, Bombus impatiens
Source: Sci Rep. 2025 Nov 26;15:42276. doi: 10.1038/s41598-025-22720-w (PMC12661002; doi:10.1038/s41598-025-22720-w)
Supplement: Supplementary file 1 — Supplementary Material 1 [file 41598_2025_22720_MOESM1_ESM.docx]

**Title**: Common herbicide impairs fertility but not survival in bumblebees, *Bombus impatiens*

**Authors:** Andrew F. Brown^1^, Pierre Giovenazzo^2^, Marilene Paillard^2^Andrée Rousseau^2^, Verena Strobl^1^, Annette Van Oysteayn^4^, Peter Neumann^1,3^, Lars Straub^1,5^

**Affiliations:**^1^ Institute of Bee Health, Vetsuisse Faculty, University of Bern, Bern, Switzerland

^2^ Department of Biology, Vachon Pavillon, Université Laval, Québec, QC G1V 0A6, Canada

^3^ Centre de recherche en sciences animales de Deschambault (CRSAD), Qc, Canada

^4^ Swiss Bee Research Center, Agroscope, Bern, Switzerland

^5^ Biobest Group NV , Research and Development, Westerlo, Belgium

^6^ Centre for Ecology, Evolution, and Behaviour, Department of Biological Sciences, Royal Holloway University of London, Egham, United Kingdom

*** Corresponding author:** [andrew.brown@unibe.ch](mailto:andrew.brown@unibe.ch)

**Keywords:** agrochemicals, biodiversity, fitness, risk assessments, reproductive toxicology, sustainability

**Figures**

**Figure S1**: Photo of male bumblebees (Bombus impatiens) kept in either individual **(A)** or hoarding cages of six bees per cage **(B)**. Arrows on figure A indicate both sucrose and pollen feeders providing ad libitum feed.


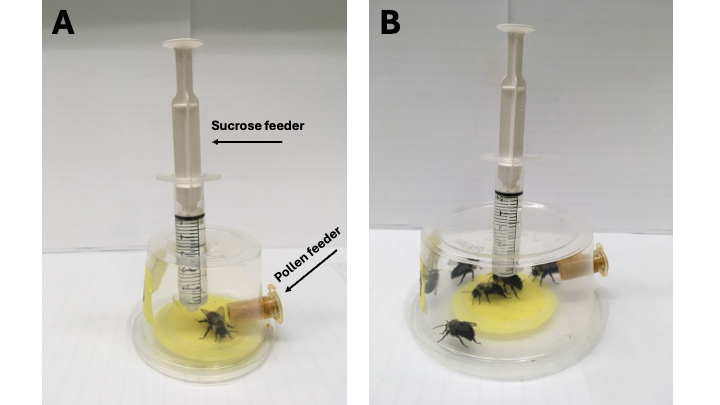


**Figure S2:** Consumption of male bumblebees (Bombus impatiens) subject to two treatments: control or herbicide, maintained in either individual (N = 50 / treament) or hoarding cages (N = 54 / treatment). Minimum, maximum, quartiles, and individual data points are displayed. The ** denotes statistical difference (P < 0.05).


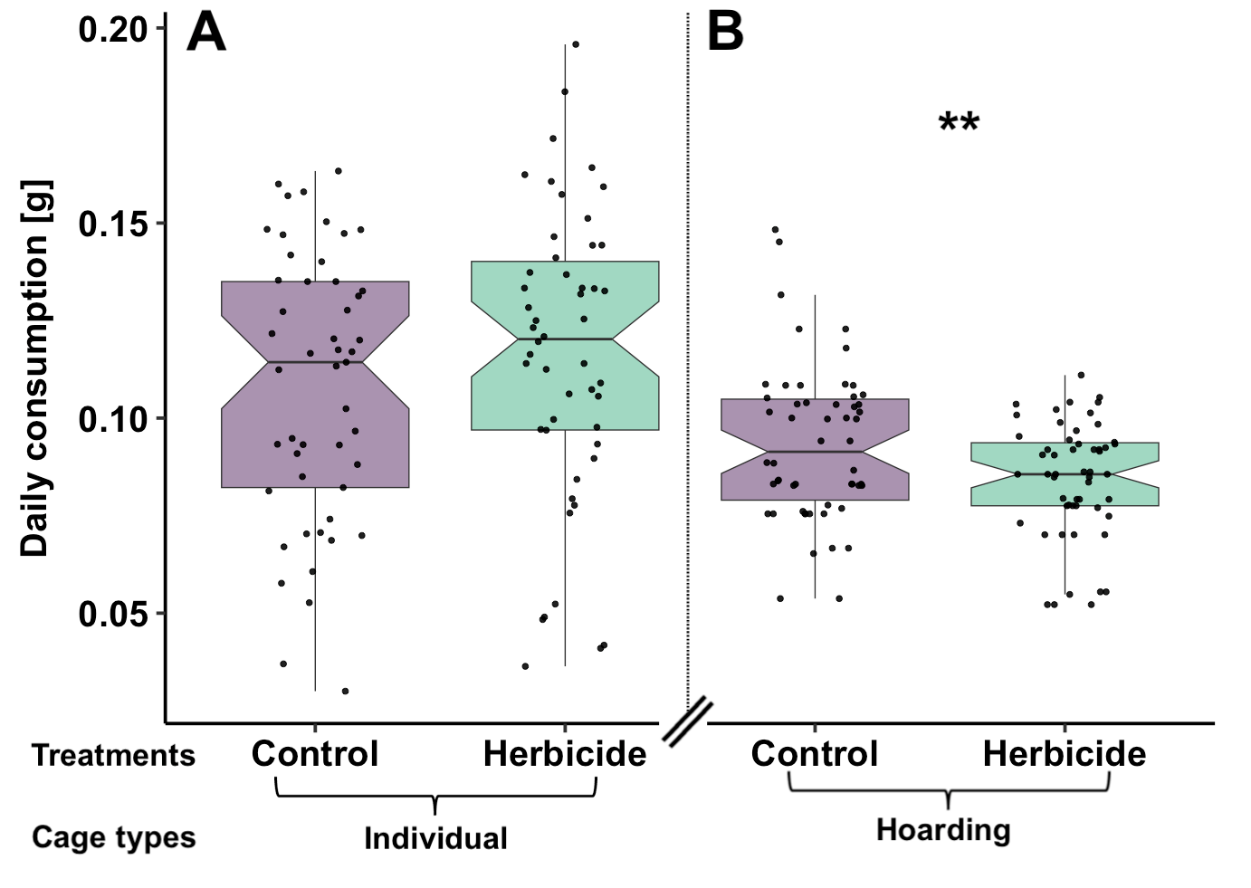

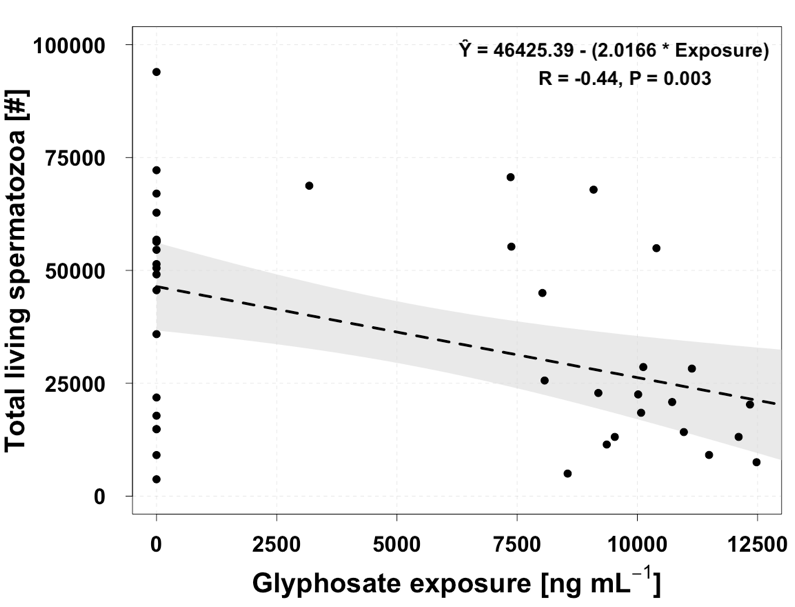


**Figure S3**: Scatterplot of total living spermatozoa [#] after 10-days of glyphosate exposure (N = 19 control, N = 22 herbicide, N = 41 total). Individual points, best fit regression line, shaded 95% confidence interval , linear prediction equation, Pearson R coefficient and P-value are displayed. The fixed estimated (-2.0166) and negative R coefficient (-0.44) show a significant decrease in total living spermatozoa as glyphosate exposure increases.

**Tables**

**Table S1**: Summary statistics of sucrose consumption of Bombus impatiens kept subject to two treatments: control and herbicide, kept in individual and hoarding. Values are in milligrams (mg). Means, std. deviations, and 95% CI reported.

| **Treatment** | **Mean (mg)** | **Std. deviation (mg)** | **95% CI ±** |
| --- | --- | --- | --- |
| Control (single) | 0.1081 | 0.0344 | 0.0096 |
| Herbicide (single) | 0.1161 | 0.0378 | 0.0104 |
|  |  |  |  |
| Control (hoarding) | 0.0933 | 0.0199 | 0.0053 |
| Herbicide (hoarding) | 0.0843 | 0.0148 | 0.0039 |

**Table S2**: Linear model summary of Total Living Sperm ~ Treatment. Fixed factors, estimates, standard error, t-values, and Pvalues are provided.

| **Variable** | **Estimate** | **Std. Error** | **T value** | **P value** | **Exposure Time** |
| --- | --- | --- | --- | --- | --- |
| (Intercept) | 55212 | 9756 | 5.66 | **<0.001** | 3 days |
| GBH | 10338 | 13506 | 0.765 | 0.448 | 3 days |
|  |  |  |  |  |  |
| (Intercept) | 43938 | 5238 | 8.388 | **<0.001** | 10 days |
| GBH | -15207 | 7151 | -2.127 | **0.0398** | 10 days |

**Supplementary Methods**

*Spermatozoa sample preparation.*

Individuals were briefly anaesthetized using CO_2,_ pinned to a wax plate, and dissected. Spermatozoa samples were collected from live bees following a modified protocol from Baer and Schmid-Hempel^35^. Here, the entire male genitalia, including the granular gland, accessory gland, vesical seminalis and testis were removed from each male, placed in a 1.5 mL Eppendorf® tube containing 250 μL Kiev+ buffer, and gently crushed to form a diluted spermatozoa stock solution. Immediately following homogenization, a 50 μL aliquot of the spermatozoa stock solution was set aside in a separate 1.5 mL Eppendorf® tube for analyses of spermatozoa viability (proportion of living spermatozoa). Spermatozoa viability was quantified according to established protocols^1^. Briefly, each sample was diluted with 50 μL of Kiev+ buffer before 1 μL of propidium iodide (PI) solution (1 mg mL^−1^) and 5 μL of Syber green (0.5 mg mL^−1^) (both Sigma-Aldrich, UK) were added to the suspension. The samples were then incubated for 10 min in complete darkness at 35°C and then gently vortexed. Ten μL were viewed at 400× magnification using fluorescent microscopy (Olympus BX41, Switzerland). Ten visual fields were randomly selected for each sample to quantify living and dead spermatozoa, and an average value was then calculated across these fields. Spermatozoa counts were performed by adding 50 μL of stock spermatozoa solution diluted in 50 μL Kiev+ buffer (1:1 dilution) in a 1.5 mL Eppendorf® tube. The spermatozoa density was then measured using a Neubauer counting chamber under light microscopy (Thermo Fischer Scientific, USA). The final spermatozoa density was quantified by applying the following calculation^2^. Total spermatozoa quantity (250 μL) = average number of spermatozoa counted in two Neubauer counting chambers x conversion factor (50,000)/ (250 μL/1000 μL).

1: Wegener, J. *et al.* In vivo validation of in vitro quality tests for cryopreserved honey bee semen. *Cryobiology* **65**, 126-131(2012).

2: Strobl, V. *et al.* Not every sperm counts: Male fertility in solitary bees, *Osmia cornuta*. *PLoS ONE* **14**, (2019).
